# Revisiting the patriarchal bargain: The intergenerational power dynamics of household money management in rural Nepal

**DOI:** 10.1016/j.worlddev.2018.08.002

**Published:** 2018-12

**Authors:** Lu Gram, Jolene Skordis-Worrall, Jenevieve Mannell, Dharma S. Manandhar, Naomi Saville, Joanna Morrison

**Affiliations:** Institute for Global Health, University College London, 30 Guilford Street, WC1N 1EH, United Kingdom

**Keywords:** Empowerment, Power, Agency, Intergenerational relations, Household finances, Money management

## Abstract

Although power struggles between daughters-in-law and mothers-in-law in the South Asian household remain an enduring theme of feminist scholarship, current policy discourse on ‘women’s economic empowerment’ in the Global South tends to focus on married women’s power over their husband; this neglects intergenerational power dynamics. The aim of this study was to describe and analyze the processes involved in young, married women’s negotiations of control over cash inside the extended household in a contemporary rural Nepali setting. We conducted a grounded theory study of 42 households from the Plains of Nepal. Our study uncovered multiple ways in which junior wives and husbands in the extended household became secret allies in seeking financial autonomy from the rule of the mother-in-law to the wife. This included secretly saving up for a household separation from the in-laws. We argue these secret financial strategies constitute a means for junior couples to renegotiate the terms of Kandiyoti’s (1988) ‘patriarchal bargain’ wherein junior wives traditionally had to accept subservience to their husband and mother-in-law in exchange for economic security and eventual authority over their own daughters-in-law. Researchers, activists and policy-makers concerned with women’s economic empowerment in comparable contexts should consider the impact of intergenerational power relations on women’s control over cash.

## Highlights

• We analyzed the intergenerational power dynamics of money management in rural households in contemporary Nepal.
• We found that junior wives and husbands often became secret allies in seeking financial autonomy from their in-laws.
• Intergenerational power relations may be just as important as male-female power relations for women’s economic empowerment.

## 1 Introduction

Women’s empowerment is widely recognized as an important policy priority (UN General Assembly, 2015), a key component of global strategies to promote health (Every Women Every Child, 2017) and combat poverty (World Bank, 2012), and an integral part of the development process (Fielding & Lepine, 2017). Women’s economic empowerment, a sub-component of their wider empowerment, can be defined as ‘the process which increases women’s real power over economic decisions that influence their lives and priorities in society’ (Tornqvist & Schmitz, 2009). Economic development programs and policies to promote women’s economic empowerment in low-resource countries often seek to reduce sex disparities in access to cash, savings, credit and income (Buvinic & Furst-Nichols, 2014) based on the theoretical justification that improved market opportunities for women outside the household ought to strengthen their bargaining power inside the household (Doss, 2013).

However, an exclusive focus on sex disparities in economic outcomes is problematic, as women’s economic empowerment is not only constrained by power relations between men and women, but also by oppression of women by women (Cornwall, 2007, Vera-Sanso, 2008). In particular, a large and well-established body of anthropological literature has analyzed the central role of intergenerational power struggles between daughters-in-law and mothers-in-law in shaping household dynamics in South Asia (Allendorf, 2017, Bennett, 1983, Bennett, 1992, Kandiyoti, 1988, Mandelbaum, 1993, Minturn and Kapoor, 1993, Vera-Sanso, 1999). Nonetheless, relatively little of this literature has applied the lens of intergenerational power struggle to analyzing women’s *economic* empowerment.

We present findings from a grounded theory study on the intergenerational power dynamics of money management within rural Nepali households. These power dynamics remain a critical area of concern for feminist researchers (Bennett, 2013, Dwyer and Bruce, 1988, Young et al., 1981), because women’s *control* over key economic resources is more important to their agency than mere ownership over resources or participation in income-generation (Kabeer, 1999). The aim of this study is to describe and analyze the processes involved in young, married women’s negotiations of control over cash inside the extended household in a contemporary rural Nepali setting.^1^ Our findings extend existing theories of women’s economic empowerment by examining the complex processes of negotiation, contestation and subversion (Kabeer, 1999) involved in asserting power over cash. We show that junior wives and their husbands often have common cause for working together rather than opposing one another in the joint household, which leads to husbands being supportive of their wives’ financial autonomy, while mothers-in-law are opposed to it. We argue that intergenerational power relations may be just as important as male-female power relations in determining women’s control over cash in our context.

## 2 Literature review

Kandiyoti (1988) famously posited that women living in patrilocal-patrilineal households in the ‘patriarchal belt’ (Caldwell, 1978) stretching from North Africa over the Middle East to South and East Asia ‘strategize within a set of concrete constraints [termed] *the patriarchal bargain*'^2^ (p. 275). According to this ‘bargain’, young brides are made to accept deprivation, hardship and subservience to their husband and mother-in-law in exchange for economic security through their husband and married sons and control over their own daughters-in-law in due time. As married men become simultaneously responsible for the economic well-being of both wife and mother, a conflict of interest emerges between mother-in-law and daughter-in-law over the allegiance of the same man. However, Kandiyoti (1988) also emphasized that such bargains are neither uncontested nor immutable, but ‘susceptible to historical transformations that open up new areas of struggle and renegotiation of the relations between genders’ (p. 275).

Indeed, as indicated in Lynn Bennett’s (1983) ethnography of upper-caste Hindu households in Nepal, a parallel bargain is often made by junior males who exchange deference to their father and elder brothers for the opportunity to succeed them at their time of death or senility. Junior males can opt out of this bargain by asking for a ‘household separation’, i.e. a partition of the family estate and the relocation of the junior couple into a new place of residence. However, such a demand forfeits the junior couple’s right to economic support from the extended household and is widely seen as a betrayal of the Hindu ideal of the joint household. As put by Bennett (1983):

‘[T]he point is that every young married couple … face a choice of strategies. They may accept the [patrilineal] ideal and wait to succeed their elders after a prolonged period of dependency. Or they may reject [patrilineal] authority and establish their own separate household. It is this choice which lies at the root of the proverbial tension between mothers and daughters-in-law and the similar but less admissible tension between fathers and sons.’ (p. 186)

Similar observations have been reported throughout South Asia in both Hindu and Muslim populations (Foster, 1993, Mandelbaum, 1993, Minturn and Kapoor, 1993, Shah, 1988). Given the widely known nature of such conflicts, it is perhaps surprising that existing literature in South Asia or comparable contexts on women’s economic empowerment has paid relatively little attention to the role of intergenerational power dynamics in shaping women’s control over cash inside the household (Vera-Sanso, 2008). A large body of work in economic bargaining theory^3^ has explored women’s bargaining power in their spousal relationship (Agarwal, 1997, Doss, 2013, Richards et al., 2013), but, to our knowledge, few empirical studies have analyzed women’s bargaining power vis-à-vis their in-laws or their own adult offspring. Similarly, anthropological studies of household management of money in Bangladesh (Kabeer, 1997, Kibria, 1995), Turkey (Eroglu, 2009) and Egypt (Hoodfar, 1997) have focused on women’s negotiations over money in their spousal relationship. Chowbey’s (2017) study of South Asian women’s strategies for coping with economic abuse in Britain, India and Pakistan analyzed men’s abuse of power over women, which was ascribed to ‘patriarchal norms such as cultural constructions of money and masculinity’ (p. 466). Vera-Sanso’s work on urban families in Tamil Nadu addressed issues of female labor force participation and filial old-age support, but did not explore the power dynamics between junior and senior women over the use of cash inside the extended household (Vera-Sanso, 2000, Vera-Sanso, 2005, Vera-Sanso, 2012).

A notable exception is the work by Singh and Bhandari, 2012, Singh, 1996, Singh, 2006 who pointed out that, within the joint Indian household, ‘the emphasis is not on the relative decision-making power of the husband versus wife. Decision-making power rests usually with the husband’s parents, but changes as the daughter-in-law gains more status and power in the household’ (p. 383) (Singh, 2006). Urban middle- and upper-class parents often have joint bank accounts with their adult children and Indian law recognizes the Hindu Undivided Family as a legal construct for tax purposes (Singh, 1996). In a study of money management in 27 urban middle-income Indian households, Singh and Bhandari (2012) reported that male household members predominantly had the final say on major financial decisions in both joint and nuclear households. However, the day-to-day management of household monies was almost exclusively carried out by women in nuclear households, while the same task was chiefly the responsibility of men in joint households.

Nonetheless, Singh and Bhandari (2012) did not provide disaggregated data on decision-making power in joint households between mothers-in-law and daughters-in-law or between fathers and sons. Their study was also primarily descriptive and offered few explanations for the observed distribution of power apart from an ‘ideology of male dominance and male control’ (p. 60). In line with previous anthropological studies on money management in South Asia, Singh and Bhandari (2012) also only sampled urban households. Thus, there is a need for more research on money management in rural populations where household economies are more reliant on subsistence production (Whitehead, 1984).

In the next sections, we will present the study context followed by the data collection and analysis methods for our study. Afterwards, we present our study findings and conclude with a discussion of the theoretical and practical implications of our findings.

## 3 Methods

### 3.1 Study context

The research reported in this article was part of a large-scale cluster randomized controlled trial in the Mahottari and Dhanusha districts of Nepal, bordering Bihar state in India (Saville et al., 2016). The primary aim of the trial was to evaluate the impact of participatory women’s groups either alone or combined with food or cash transfers, on birth weight. This sub-study was conducted as part of a wider effort to understand the household context underlying rural, pregnant women’s ability to control monthly unconditional cash transfers that were provided to them in the trial. Mother and Infant Research Activities (MIRA), a Nepalese research NGO, ran trial activities on the ground from 13 Feb 2014 until 10 October 2015, in collaboration with the UN World Food Program and Save the Children. The full design of the trial has been reported elsewhere (Saville et al., 2016).

For the purposes of our current study it is important to understand the socio-cultural context of the trial. The total population of the districts is 1.4 million, 87% are Hindu and 11% Muslim. Male foreign labor migration is widespread with top destinations being India, Qatar, Kuwait, United Arab Emirates and Malaysia (Ministry of Labour and Migration, 2014). A study of local pregnant women aged 15–45, found that 36% of women’s husbands had migrated abroad, mostly to Middle Eastern countries (61.5%), India (27.9%) or Malaysia (8.8%) (Gram et al., 2018). The same survey also found that 87% of these women lived in joint households, 9% lived separately from their in-laws, and 4% of households had experienced the death of the mother-in-law (Gram et al., 2018).

The local language and majority culture is Maithili. As the second-largest language in Nepal, 3 million native Maithili speakers live in Nepal (Central Bureau of Statistics, 2012). 30 million live in India (Lewis, 2016). Maithili culture shares many similarities to cultures in the ‘purdah belt’ (Mandelbaum, 1993) comprising North India, Bangladesh and Pakistan including the predominance of the patrilocal-patrilineal household (Agarwal, 1994). A household hierarchy operates where higher-ranking members are ‘guardians’ of lower-ranking members, with attendant duties to supervise their conduct and ensure their physical and social well-being (Morrison et al., 2018). Guardianship is determined by age and gender, thus mothers-in-law are guardians of daughters-in-law and fathers are guardians of their sons, but other household members can take over the guardianship role, if the existing guardian dies or reaches senility.

Pro-natalist social norms put considerable pressure on newly married women to conceive of sons as quickly as possible. Families where male family members work abroad often ensure newly married daughters-in-law become pregnant before their husband leaves the country. Sons are preferred over daughters to generate labor and dowry income (Bennett, 1983). Women failing their reproductive ‘obligations’ to become pregnant or give birth to a son may be perceived to constitute a drain on the household economy and risk abandonment in the early years of their marriage (Clarke et al., 2014). Mothers without sons have been found to experience extreme psychological distress, as they are taunted by their guardians and neighbors for ‘never having had a son in their womb’/*niputar* and are believed to suffer from a curse (Clarke et al., 2014).

Female seclusion norms stigmatize married women’s free movement in public spaces and prevent newly married daughters-in-law from leaving the house (Acharya and Bennett, 1983, Morrison et al., 2018). Local women and men use the colloquialism, ‘the frog in the well’/*inaar ke beng*, to describe newly married women’s sense of being trapped in the house with few opportunities for escape (Gram et al., 2018). Physically leaving the house does not in itself constitute a breach of social norms, but women who are perceived as loitering without a purpose provoke connotations of sexual availability and impropriety (Gram, 2018). Women who wish to physically leave the confines of the home usually require explicit permission from a senior family member and the provision of an escort to signal that they have entered the public sphere for a legitimate purpose. Women living in nuclear families have greater mobility, as their *de facto* status as the eldest woman of their household justifies their public presence.

### 3.2 Data collection

We used a grounded theory methodology (Corbin & Strauss, 2008) to develop and extend existing theories of household money management for our local context. Our sampling frame and topic guides evolved over time, to explore emergent themes over the course of data collection. This involved an iterative loop of conducting interviews, transcribing audio recordings, analyzing data, focusing topic guides and sampling new respondents based on prior data analysis. The purpose of this process was to maximize our chances of inductively developing a rich, nuanced theory with a coherent logic where all the theoretical categories were connected to one another and no major explanatory gaps were left. Over the course of this cycle, we asked male and female respondents about household management of money, attitudes to women’s economic autonomy, support and conflict in the household, separation from the joint household, family honor and friendships, experiences with male migration, and women’s experiences of pregnancy.

At the time of the study, districts in Nepal were divided into Village Development Committees (VDCs). Table 1 provides a short description of the VDCs where interviews took place. All the VDCs belonged to the cash transfer arm of the wider trial. In this paper, respondent quotes are labelled with a pseudonym for the VDC where interviews were conducted and a serial number to maintain respondent anonymity.

**Table 1:** Characteristics of VDCs where interviews were held.

VDC	% Dalit	% Muslim	% Women with no education	% Below median regional wealth levels	Travel time to district headquarters by motorbike	Travel time to Indian border by motorbike
Kote	31.6%	3.7%	63.1%	73.5%	2–4 h	>1 h
Jamal	13.1%	49.9%	78.9%	46.9%	1–2 h	>1 h
Sahku	19.3%	1.6%	48.4%	36.8%	30 mins	30 mins
Lalit	10.9%	0.3%	60.4%	61.2%	2–4 h	>1 h
Dahak	22.5%	10.2%	63.6%	60.5%	30 mins	45 mins

At the time of the study, districts in Nepal were divided into Village Development Committees (VDCs). All VDCs belonged to the cash transfer arm of the trial. Pseudonyms have been created to serve as VDC names to ensure the confidentiality and anonymity of the interviewees. All % have been estimated from surveillance data in the wider randomized controlled trial. Wealth levels were derived using an asset index (Filmer & Pritchett, 2001). Travel times to the Indian border indicate links to foreign trade, while travel times to district headquarters indicate links to domestic trade.

Table 2 shows the characteristics of female respondents. We conducted semi-structured interviews with 22 currently or recently pregnant daughters-in-law and their guardians. We exclusively sampled currently or recently pregnant daughters-in-law, as this was the target population of the wider trial. The guardians were usually mothers-in-law (n = 15), but were sometimes elder sisters-in-law (n = 3) in cases where the mother-in-law had died. We sampled daughters-in-law to ensure variation in geographical region, caste, socio-economic status, education, nuclear versus joint household, and husband’s migrant status. Due to low levels of ethnic variability in our geographical context, we did not sample based on ethnicity. We did not explicitly sample guardians based on any criteria, but instead interviewed any consenting guardian of a sampled daughter-in-law.

**Table 2:** Female respondent characteristics.

	Household characteristics					Mother-in-law/Elder sister-in-law		Daughter-in-law		
Serial no.	VDC	Caste	SES	Nuclear/Joint household	Husband working abroad?	Family relation	Grades passed	Age	Grades passed	Pregnant?
1	Sahku	Dalit	Middle	Joint	No	Mother-in-law	0	20	7	No, baby 5 months
2	Sahku	Dalit	Low	Nuclear	Yes, Middle East	Mother-in-law	0	25	0	No, baby 7 months
3	Sahku	Brahmin	High	Joint	No	Sister-in-law	7	21	8	No, baby 6 months
4	Sahku	Brahmin	High	Joint	No	Mother-in-law	0	21	8	No, baby 4 months
5	Sahku	Middle Madhesi	Low	Nuclear	No	Mother-in-law	0	21	7	No, baby 1.5 months
6	Sahku	Middle Madhesi	Middle	Joint	No	Mother-in-law	0	21	5	Yes, 7 months
7	Sahku	Middle Madhesi	High	Joint	Yes Middle East	Mother-in-law	0	24	8	No, baby 5 months
8	Sahku	Brahmin	High	Joint	No	Mother-in-law	7	20	12	No, age of baby unknown
9	Sahku	Dalit	Low	Joint	No	Mother-in-law	0	30	0	No, baby 5 months
10	Sahku	Middle Madhesi	High	Nuclear	No	MIL refused consent		21	0	Yes, 8 months
1	Kote	Dalit	Middle	Joint	No	Mother-in-law	0	20	14	Yes, 8 months
2	Kote	Dalit	Low	Nuclear	No	Equipment failure		25	5	No, baby 5 months
3	Kote	Dalit	Low	Joint	Yes, Middle East	Sister-in-law	0	18	6	No, baby 1 month
4	Kote	Dalit	Low	Joint	Yes	Mother-in-law	0	25	8	No, age of baby unknown
5	Kote	Dalit	Middle	Nuclear	Yes, Middle East	Mother-in-law	0	25	0	Yes, 7 months
6	Kote	Dalit	Middle	Joint	No	Mother-in-law	0	20	0	No, baby 1 month
7	Kote	Dalit	Middle	Joint	No	Mother-in-law	0	22	12	No, baby 7 months
8	Kote	Middle Madhesi	Low	Joint	Deceased	Mother-in-law	0	20	3	No, baby 2 months
1	Jamal	Dalit	Middle	Joint	Yes	MIL refused consent	20	0		No, baby 4 months
2	Jamal	Middle Madhesi	High	Joint	Yes, Malaysia	Mother-in-law	0	25	8	No, baby 6 months
3	Jamal	Dalit	Low	Nuclear	No	Sister-in-law	0	25	8	No, baby 3 months
4	Jamal	Dalit	Low	Joint	Yes, Delhi	MIL refused consent	17	5		No, age of baby unknown

Socioeconomic status was appraised by local informants and adjusted based on interview data in case of discrepancies. Destination country of migration was not reported for respondent 4, Kote and 1, Jamal. Ages for mothers-in-law/elder sisters-in-law not provided, as mothers-in-law had difficulties reporting their age exactly, although it ranged from 50 to 80 years. All daughters-in-law gave consent. All respondents were of Hindu faith.

Pilot interviews indicated mothers-in-law were reluctant to let us interview their daughter-in-law in private, while daughters-in-law felt intimidated by the prospect of discussing household matters in front of their mother-in-law. We therefore conducted concurrent but separate interviews with daughters-in-law and their guardian, in two separate locations around the household. While the daughter-in-law was interviewed in her room, the guardian was simultaneously interviewed alone in an outside location, such as under a tree or in a nearby field. This ensured a high degree of privacy for the daughter-in-law, as male household members were at work and other female household members may have presumed our interview with the mother-in-law was more significant and of greater interest. A similar design is sometimes used in Western settings to separate married women from their husbands during interviews (Hertz, 1995). None of the daughters-in-law refused consent, but three mothers-in-law refused consent because they did not have the time. In these cases, we still interviewed their daughter-in-law alone. The recording of one mother-in-law was lost due to equipment failure.

We also interviewed 20 husbands to explore male perspectives on household finances. This was necessary to obtain a complete picture of household finances, as female household members were often excluded from discussions involving male household members and Maithili sexual taboos strongly prohibited casual communication between female household members and their elder male in-laws (Gram, 2018). Table 3 shows the male respondents’ characteristics and the pregnancy status of their wives. All husbands were married to currently or recently pregnant women. One husband’s wife had an abortion, one suffered a miscarriage and one recently experienced the death of their newborn. The husbands were also sampled to ensure variation in geographical region, caste, socio-economic status, education, and nuclear versus joint household. The husbands were all interviewed alone in an outside location.

**Table 3:** Male respondent characteristics.

Serial no.	VDC	Caste	SES	Nuclear/joint household	Age	Grades passed	Interview taken?	Wife pregnant?
1	Dahak	Middle Madhesi	II	Nuclear	40	0	Yes	No, baby 1 month old
2	Dahak	Middle Madhesi	IV	Joint	30	9	No, consent refusal	No, baby 18 days’ old
3	Dahak	Middle Madhesi	IV	Nuclear	26	4	Yes	No, baby 17 days’ old
4	-Dahak	Middle Madhesi	V	Joint	28	8	Yes	No, baby 2 months old
5	Dahak	Dalit	I	Nuclear	30	0	No, consent refusal	No, baby 1 month old
6	Dahak	Dalit	I	Joint	?	0	Yes	No, baby 6 months old
7	Dahak	Middle Madhesi	IV	Joint	22	11	Yes	No, baby 1 month old
8	Dahak	Middle Madhesi	I	Joint	23	4	Yes	No, wife had an abortion
9	Dahak	Middle Madhesi	V	Joint	28	13	No, consent refusal	Unknown
10	Dahak	Muslim	I	Joint	26	0	No, moved abroad	Unknown
11	Dahak	Dalit	I	?	?	0	No, consent refusal	Unknown
1	Lalit	Middle Madhesi	III	Joint	23	6	Yes	No, baby 14 days’ old
2	Lalit	Middle Madhesi	V	Joint	38	8	Yes	No, baby 1 month old
3	Lalit	Middle Madhesi	I	Joint	32	6	Yes	No, baby 1 month old
4	Lalit	Middle Madhesi	III	Joint	31	0	Yes	Yes
5	Lalit	Middle Madhesi	II	Nuclear	29	0	Yes	Yes
6	Lalit	Middle Madhesi	IV	Nuclear	41	8	Yes	Yes
7	Lalit	Middle Madhesi	III	Joint	24	11	Yes	No, baby 1 month old
8	Lalit	Dalit	II	Joint	24	5	No, consent refusal	Unknown
9	Lalit	Middle Madhesi	V	Joint	26	10	No, moved abroad	Unknown
1	Jamal	Middle Madhesi	V	Joint	28	6	Yes	No, baby 1 month old
2	Jamal	Middle Madhesi	III	Nuclear	32	5	Yes	No, baby 1 month old
3	Jamal	Dalit	I	Joint	25	0	Yes	No, baby 1 day old
4	Jamal	Muslim	III	Joint	35	0	Yes	Yes
5	Jamal	Middle Madhesi	III	Joint	28	0	Yes	No, wife suffered miscarriage
6	Jamal	Muslim	III	Nuclear	44	0	Yes	No, baby died
7	Jamal	Middle Madhesi	V	Joint	25	7	Yes	No, baby 2 months old
8	Jamal	Muslim	I	Joint	40	0	No, consent refusal	Unknown

SES reflects socioeconomic quintiles calculated using an asset index (Filmer & Pritchett, 2001) from surveillance data from the wider randomized controlled trial, I = lowest socioeconomic status, V = highest. Husbands 6 and 11 in Dahak VDC did not provide information on their age. Husband 11 in Dahak did not provide information on household structure.

To interview the husbands, we chose not to revisit the original households where we had interviewed female household members, as this might impose excessive time burdens on households in the context of repeated quantitative surveys on nutrition and health from the wider trial. Even so, five men refused to participate because they did not have time. One husband expressed offence at being asked questions that ‘should be asked of women only’. Husbands are often more challenging to interview than wives due to their need to leave for work early in the morning and spend time with the family in the evening. By comparison, young, married women’s restricted mobility facilitates consent. The moderately high refusal rate of husbands (5 out of 28) presents a concern that consenting husbands may differ from non-consenting husbands, for example by having more cooperative spousal relations. We addressed this by triangulating our findings from the husbands with those of the wives and their guardians who were sampled from entirely separate households.

All interviews with both women and men were audio recorded and lasted one to two hours. At the end of the data collection process, we were approaching saturation as new analytical insights arising from further interviews became increasingly rare. Nonetheless, emergent hypotheses about the influence of pregnancy and childbearing on women’s control over cash could have been explored in greater depth by sampling married women who were neither pregnant nor had young children.

### 3.3 Data analysis

Native Maithili speakers directly transcribed and translated the data from the audio recordings into written English. The first author (LG) performed the coding and analysis of the translated data. For each transcript, reflective notes were written using analytical tools from grounded theory followed by open and selective coding. To reduce cultural bias, stimulate reflective analysis and ensure analytic rigor, LG discussed findings and interpretations of the data with interviewers, translators and co-authors throughout data collection and analysis. At the end of the analysis process, all the ideas, concepts and relationships were integrated into a single ‘storyline’ to check for logical consistency.

## 4 Results

### 4.1 Financial management in the joint household

The overarching goal of financial management in joint Maithili households was referred to by respondents as ‘fulfilling the needs of family members’. This was primarily realized through an ideology of ‘financial guardianship’, where a key person, the ‘financial guardian’,^4^ was entitled to physically receive and manage most household income. The financial guardian was not always the household head. The household head might have the final say over financial matters and make decisions with broad economic impacts such as house construction or business investments. By comparison, the financial guardian physically received the household income, managed day-to-day expenditure decisions, allocated purchased goods among household members, and coordinated borrowing, lending and saving operations. Indeed, many husbands explained that naturally no-one saved up money separately, since they all lived together in a joint household. One husband even commented that centrally managed household income was one of the defining characteristics of a joint household:

Husband: *If every earner keeps his income for himself then there is no need of guardianship and the household will automatically break up [into smaller units]*.

husband 4, Dahak

Both mothers-in-law and fathers-in-law were seen to be fit for financial guardianship due to their high rank in the household, their standing and social networks in the community and their experience with the village economy. Patrilineal inheritance customs meant they were the only household members who could credibly offer moneylenders the family estate as collateral for the large loans necessary to sponsor foreign labor migration, marriage of daughters, or house construction projects. However, mothers-in-law were often preferred to fathers-in-law as financial guardians for three key reasons: First, in a context where only the wealthiest households had a bank account, women needed to know the physical location of valuable household assets to safeguard them from theft while male household members were out for work during the day. This made female household members more ‘natural’ candidates for physically receiving and guarding household cash. One NGO staff member explained in English that ‘*in Nepal, women are just like a bank*’. Second, widely shared gendered expectations that it was the duty of women to handle matters of food preparation and serving led most husbands to avoid any involvement in the day-to-day budgeting of (mostly food-related) expenditures. Finally, many husbands felt that it was ‘the nature of women’ [husband 3, Jamal] to save money; thus ‘sensible’ husbands let female household members physically control the household money to prevent it from being wasted on expensive meat or alcohol.

Regardless of how tasks and responsibilities were shared between mothers-in-law and fathers-in-law,^5^ the overall system invariably left junior husbands and wives disempowered as they neither qualified for financial guardianship nor for household headship. Thus, households needed to reach a compromise between the need for central coordination and the need for financial autonomy. Indeed, a counter-ideology of ‘personal property’ existed where individuals had a right/*haq* to their own private money, particularly earned money, which they could dispose of however they liked. Household members sometimes had individual personal lockers in which they could store private money, and young brides received a trunk/*peti* with a secure lock upon marriage from their natal family where they could store their private possessions. Households allowed male earners to keep a small portion of their income for personal usage, called ‘pocket money’ (*sic*), while requiring them to hand over the rest to the financial guardian. Female income earners, on the other hand, had to hand over the full amount to the financial guardian, whether they were daughters-in-law or mothers-in-law.

We found a few exceptions to the predominant pattern of money management. In one household, income-earners alternated in contributing to the household fund [daughter-in-law 2, Jamal]. The daughter-in-law lived with her husband, brother-in-law, co-daughter-in-law, mother-in-law and father-in-law. Every month, either her husband or her brother-in-law were responsible for paying the joint expenses of the whole household. In months, where neither could afford this, the mother-in-law and father-in-law paid out. This household was a highly educated, well-connected family whose junior male and female members had a Bachelor’s Degree level or were studying for one, and whose youngest children attended boarding school in Kathmandu. As such this household was highly unusual in our sample. In another household [daughter-in-law 7, Kote], the mother-in-law became ostracized from her family and sent to live in a small hut after her husband, the main income earner of the household, died. Instead, the oldest daughter-in-law took over as financial guardian in the household. However, money management practices did not vary systematically in our sample according to caste, aggregate household wealth, the relative size of household members’ earnings or their relative levels of education. Money management practices also did not vary systematically by women’s pregnancy status.

### 4.2 Financial risks for daughters-in-law in the joint household

Female seclusion norms precluded daughters-in-law in joint households from visiting a market on her own, leaving them dependent on other household members to buy what they needed.^6^ Door-to-door salesmen provided regular opportunities to buy food, clothes, kitchenware and cosmetics, and daughters-in-law sometimes sent their children out to purchase from the local village shop. However, prices were higher for such purchases than at the marketplace. If daughters-in-law wanted to procure items from the market they would have to ask their financial guardian for money to give a senior female or male household member to take with them to the market. Such requests needed to be justified on a case-by-case basis.

Mothers-in-law were significantly less restrained by mobility norms; they frequently took out loans, shopped at the market or participated in savings and credit groups as part of their role as financial guardians. However, allowing daughters-in-law to have any personal cash was perceived by mothers-in-law as a major concession of power, as it was their responsibility and privilege to regulate daily financial transactions. Thus, the needs of daughters-in-law should only be met through purchases that had been approved by them:

Interviewer: *Do you allow your daughter-in-law to have her own savings?*

Mother-in-law: *No, I do not allow her…Why does she need to have her own money? Presently, we are there to do everything for her. She will have her own money when the appropriate time comes*.

mother-in-law 4, Sahku

However, daughters-in-law often felt dissatisfied with this system. Although a few instances of genuinely caring and supportive relationships existed between daughters-in-law and mothers-in-law,^7^ almost all daughters-in-law felt ignored and excluded in the household, as they held little to no right to participate in financial decisions. Several daughters-in-law explained that any attempt to even ask about the prices of goods would be seen by their mother-in-law as challenging her financial authority.

Interviewer: *Since you never go to the market, do you know the price of goods in the area? Things like rice, vegetables, potato or other things?*

Daughter-in-law: *Only those people who go know the price of goods…I don’t know them*.

Interviewer: *Do you ask your family members about such prices?*

Daughter-in-law: *No, I don’t ask…[If I asked,] they might think I’m taking down a balance record of their purchases. My mother-in-law says this to me. Don’t you know the rules of the village?*

daughter-in-law 6, Kote

According to the financial norms of the joint household, daughters-in-law should be able to ask their mother-in-law whenever they needed anything. In practice, they often felt both embarrassed and humiliated for having to constantly ask for small amounts of cash. Daughters-in-law particularly worried about the ability or willingness of their in-laws to pay for their food and health care costs, and often found themselves in the difficult position of being simultaneously responsible for their own children’s welfare while being disempowered from making financial decisions on their behalf. Such experiences caused significant psychological distress:

Interviewer: *How did you feel about being left out of money matters in the household?*

Daughter-in-law: *I felt very bad. I was stressed and worried. I did not want to talk to anyone and I used to think too much. I could not even look at my children’s faces.*

*daughter-in-law 9, Sahku*

Daughter-in-law: *But these days I am suffering from a lack of money in my household. No-one can understand it except me…I can’t express my feelings to anyone else. Who do I tell my feelings to? No-one would believe me…What else can I do? I can’t do anything except cry.*

*daughter-in-law 4, Kote*

According to the patriarchal bargain, daughters-in-law were supposed to ascend the household hierarchy after the birth or marriage of their own son. In reality, the birth or marriage of a son was far from guaranteed to ensure greater control over finances for junior daughters-in-law. Some daughters-in-law who had lived with their mother-in-law for 7–15 years and had brought their own daughters-in-law into the household, still reported having no say over household financial decisions^8^ [daughter-in-law 4, Kote; daughter-in-law 9, Sahku]. When asked about the expected time taken for daughters-in-law to be involved in household money management, almost all daughters-in-law and mothers-in-law replied they would have to wait for the current financial guardian’s death or senility.

Importantly, many of the experiences reported by the daughters-in-law, both old and young, fell within contemporary definitions of economic violence (Chowbey, 2017, Fawole, 2008). This included threatening them with physical violence if they were found in possession of private cash [daughter-in-law 3, Kote], withdrawing financial support for health care in life-threatening situations [daughter-in-law 2, Kote], and withholding food as a bargaining chip in household disputes [daughter-in-law 9, Sahku]. Daughters-in-law subjected to such treatment widely condemned their household members for failing to satisfy their basic needs and distributing resources inequitably among household members.

### 4.3 Secret savings as a strategy of acquiring agency

In theory, daughters-in-law could obtain disposable cash through wage labor or investment in their own micro-enterprises with local credit. In practice, this was extremely challenging. Higher-ranking household members did not approve of daughters-in-law taking out loans on their own, as they were too junior to take out credit and imposed burdens on male income earners to repay the loans. Senior household members also worried about incurring reputational damage to the family if junior wives ventured outside and violated female seclusion norms in the community. Daughters-in-law lacked the necessary ownership of collateral and social connections to qualify for loans in the community. Hindu daughters-in-law were particularly isolated as customs of village exogamy meant they had left their friends and family behind when they married to enter a new village as a stranger.

In the case of wage labor, most daughters-in-law expressed a strong desire to earn cash.

Daughter-in-law: *I only get money when someone gives me. I am not independent. I depend on others. And if I get money then, other people may take it away and spend it. For all these reasons, I can’t just go buy things for myself at the market*.

Interviewer: *Do you want to earn money yourself?*

Daughter-in-law: *Who does not want to earn money??*

*daughter-in-law 2, Jamal*

However, daughters-in-law faced formidable barriers to doing so. The substantially higher wage rates from foreign labor migration made local income earning opportunities seem meagre in comparison;respondents reported receiving NPR 20,000–100,000 [USD 195–977] monthly per migrant earner compared to NPR 2000–10,000 [USD 20–98] per local male earner. Lack of education and physical capital led most working women to take up agricultural employment in a poorly paid labor market that devalued their contribution.

Elder sister-in-law: *We never receive cash for our work. Only men get [wages]. A man can dig mud, use a spade or construct a building, but a woman cannot do such work. Can a woman do a man’s job? No, no-one does. That’s why we never receive cash for our work*.

*elder sister-in-law 3, Kote*

Family members in all but the poorest households forbade daughters-in-law to obtain employment to avoid dishonoring male ‘breadwinners’, breaching female seclusion norms, and impeding the fulfilment of their domestic duties. As many respondents had recently given birth to a small child, they also faced prohibitions by their family members to never leave their child alone. Lack of family support also made it impractical for them to work, as many husbands, mothers-in-law and elder sisters-in-law found it near-unthinkable that they would care for the child of a daughter-in-law just so she could obtain employment.

In this context, savings held a strong advantage over employment and credit as a source of cash, since husbands and wives often had aligned incentives to allow the wife to accumulate her own savings. Junior husbands were often just as powerless to influence financial decisions as daughters-in-law due to the need to defer to either parents or older male siblings. Many husbands revealed that they were routinely left out of both major and minor financial discussions in the household and were aware that it was not their place to participate. These husbands explained that any attempt to involve themselves would be seen as a challenge to authority.

Husband: *My parents make purchases, I do not. My duty is to earn money and hand it over to them. Apart from doing this, I know nothing about money. My parents maintain our family. It is completely up to them… If they told me to buy anything, I would buy it, but otherwise I do not… I cannot even buy a packet of a salt of my own accord*.

*husband 1, Jamal*

Husbands could sometimes retain pocket money from their own earnings before handing it over to the financial guardian, but still had to ask for money from their financial guardian for larger purchases who might deny their request. In one instance, a husband wanted to send part of his earnings to pay off a debt his wife owed a relative, but his father refused, even though the husband was the main income-earner for the household [daughter-in-law 3, Kote]. In another instance, a husband was forced against his will to spend his income and his wife’s savings paying back a huge loan (worth NPR 400,000–500,000 [USD 3908–4885]) taken by his elder brother [daughter-in-law 10, Sahku]. Such husbands felt exploited by their financial guardian and either expressed a desire to formally separate from their parents or had already carried out a household separation.

Compared to the cash controlled by their elders, husbands felt they could easily access savings held by their own wives, since poor employment opportunities and female seclusion norms meant their wives depended on them to obtain more money and execute purchases anyway. In many households, junior wives accumulated *koseliya*, denoting ‘married women’s secret money’ in Maithili language,^9^ with the tacit consent of their husbands. The most common strategies for doing so included saving up gifts from one’s natal parents [daughter-in-law 7, Kote], manipulating information about prices when claiming money for expenses [daughter-in-law 1, Sahku], or selling off the household’s grain surplus in secret [mentioned by husbands 6 from Lalit, and 7 and 8 from Dahak]. One husband explained, he did not mind his wife’s secret strategies, even if they involved a degree of ‘lying’:

Husband: *To me, it’s a good thing. It does not matter if she lied to me to get money, but if she has set some koseliya aside, then one day, the money will benefit our family*.

*Husband 2, Lalit*

Junior wives put considerable pressure on their husband to directly provide them with savings from their earnings. Daughters-in-law in our sample fiercely insisted that, as much as their husbands had a duty to contribute to the extended household, they also had a duty to ensure the financial well-being of their wives and children. Caught between their duty to their wife and children and their duty to their parents and siblings, husbands often compromised by directly transferring a portion of their income to their wives in secret. When they subsequently handed over the remaining part of their income to the financial guardian, they would omit to mention the portion of their income that had been sent to their wives. For the many husbands working and living abroad in the Middle East in our context, this was accomplished by sending home remittances in the name of the wife’s natal parents. The amounts transferred through secret channels were often considerably larger than the amounts received by daughters-in-law through petitioning their in-laws. When daughters-in-law reported receiving cash from their financial guardians, the sums ranged from NPR 50–1000 [USD 0.5–9.8]. By comparison, income transferred through secret channels ranged from NPR 5000–15,000 [USD 49–147] every two or three months.

### 4.4 Preparing for a household separation

Junior couples accumulating secret savings frequently worried about the ramifications of being discovered:

Daughter-in-law: *I was worried…It could become a big issue in the household that a husband had sent secret money to his wife…They might think that, although there were lots of loans to be repaid, my husband was sending me [individual] allowances for me and my children… I did not want my sisters-in-law to feel they had been unfairly treated so I did not tell anyone*.

*daughter-in-law 5, Kote*

Extended household members also treated secret savings for the wife with extreme suspicion.^10^ One daughter-in-law was immediately accused of getting secret money from her husband when she returned home with the cash transfer provided in the intervention arm of the trial [daughter-in-law 7, Kote], while another daughter-in-law hid the money she received from her natal parents for fear her in-laws would make precisely such an allegation if they found the money [daughter-in-law 3, Kote]. Mothers-in-law also explained that if they allowed their son to provide their wives a portion of their income, they would quickly lose control over both their son and daughter-in-law [mother-in-law 4, Sahku; mother-in-law 5, Kote].

Part of the reason behind such intense suspicion was a fear that the junior couple was preparing for a household separation. Household separation carried strong economic disincentives, as the junior couple could no longer claim financial support from their in-laws and had to arrange for their own living expenses, including the cost of building a new home. Indeed, several daughters-in-law living in households where they suffered psychological and physical violence from their in-laws could not persuade their husbands to move out due to such financial considerations [daughter-in-law 9, Sahku; daughter-in-law 3, Kote]. Thus, several husbands and mothers-in-law explained that the accumulation of secret savings was often part of a wider strategy to insure the junior couple against financial risk [husband 2, Lalit; husband 6, Dahak; mother-in-law 4, Sahku; mother-in-law 5, Kote]. Separated nuclear couples also reported accumulating secret savings before their separation occurred [daughter-in-law 5, Kote; husband 8, Dahak].

Husband: *If there are several brothers in a family then everybody gives a part of their earnings to their wives to keep as secret money/koseliya… they tell them not to spend that money on anything and even tell their wives to ask for money from the guardian of the household instead of spending their own money, and not to tell anybody else about it too… they keep secret money/koseliya for themselves, for their children and for their future… so that if they would have to live separately from their family in the future then their… family should not suffer from a crisis… and they should not have to ask for money from others*.

*husband 6, Dahak*

Since sons are ‘meant’ to stay with their wives in their parents’ house for their entire lifetime in the patrilineal Maithili family, household separation is widely perceived as a traumatic breakdown of the traditional household unit. Female guardians and senior male brothers-in-law felt that husbands should never listen to their wives, because this would create rifts between household members and ultimately lead to household separation. For example, one husband blamed his brother’s wife for the decision to separate from the wider household:

Husband: *After the marriage of my second brother, a woman from another household entered our home and due to her, changes occurred that were the beginning of the destruction of our household … She told my brother to separate from the family and then he separated after one year being married.*

*husband 2, Dahak*

Nonetheless, despite all the best efforts of the extended household members, they could only do so much to prevent a determined junior couple from progressing towards separation. Separated daughters-in-law often described a newfound ability to make autonomous decisions on consumption and leisure time and an increased ability to move about in public spaces and make purchases according to their own desires.

Daughter-in-law: *I used to offer food to each family member and, while doing household work, if anything had been overlooked or some mistake had been done then they swore at me and argued with me. After I had cooked, I had to wait for all the household members to eat and then eat last myself. Now, if I want to cook, then I cook and if I want to eat, I eat*.

*daughter-in-law 5, Kote*

Interviewer: *Does anyone from your family ever tell you not to go out to buy goods or not to go to any other place? Do your family members allow you to go outside?*

Daughter-in-law: *There is no-one in the family [to tell me what to do]. I make decisions about my own household, so no-one can say things like that to me. I do everything on my own … [except] once I fell sick and my husband bought goods for the household, but when I got better, I again made purchases myself*.

*daughter-in-law 3, Jamal*

Husbands similarly found their financial autonomy increased as their parents stopped being involved in their financial and non-financial matters. Mothers-in-law, on the other hand, lamented the loss of financial support and the termination of personal relationships.

Mother-in-law: *As we live separately from each other, we are like neighbors now. Everybody knows that if your son is separated from you, then he will be like a neighbor for you, but nothing more than this … It is the rule of the community that if you live separately from your family, then you also want to take your own decisions*.

*mother-in-law 5, Kote*

## 5 Discussion

In this article, we sought to describe and analyze the complex social processes involved in young married women’s negotiations over cash in the rural Nepali household. In line with previous literature on women’s strategies for control over money (Chowbey, 2017, Eroglu, 2009, Kabeer, 1997), financial secrecy was an important means of exerting agency and resisting total economic dependency in a context where women’s dependent status served as strong deterrents to overt confrontation with other household members (Jack et al., 2010, Mandelbaum, 1993). In contrast to previous accounts, we found a central role for intergenerational power dynamics, where husbands and daughters-in-law acted as allies in a struggle for financial power against the financial guardian of the household. Junior wives and husbands who felt dissatisfied with the implementation of the patriarchal bargain in their household were led to accumulate secret savings with a possible view to eventually separating from the joint household and forming their own nuclear household. Extended household members strongly opposed this, because they benefited from being able to access the income of the junior couple and thus closely policed their access to cash, which only further strengthened incentives to accumulate secret money, potentially causing a self-reinforcing spiral. These dynamics may be important to take into account when designing policy and research into women’s economic empowerment.

Our results extend previous theories of financial secrecy, which have hitherto seen women’s covert resistance to patriarchy as ‘a curious combination of ritualistic adherence to traditional norms in public on the one hand and private mockery and manipulation of *husbands* on the other’ (emphasis mine) (Bolak, 1997, Eroglu, 2009), while ignoring intergenerational power dynamics in the joint household. For example, Eroglu (2009) listed reasons for accumulating secret money, such as preventing husbands from wasting their income, avoiding blame for misusing visible funds, or protecting their husband’s masculine pride, that might not apply in a context where daughters-in-law obtained secret money through the co-operation of their husband.

Nonetheless, our finding that the mother-in-law, rather than the father-in-law, often assumes the role of financial guardian resonated with previous studies on money management, where women in low-income nuclear families were referred to as ‘financial managers’ (Hoodfar, 1988), ‘family bankers’ (Hoodfar, 1988) or family ‘cashiers’ (Zelizer, 1989) and the ability to receive and nurture household income was found central to women’s identity (Busby, 1999). Our results also replicated Singh’s (2006) observation in urban India that senior in-laws typically held financial decision-making power over the junior couple in the joint household. That junior wives had to ask for and justify access to cash on a case-by-case basis resembled the ‘irregular dole system’ reported by Singh and Bhandari (2012). Previous quantitative studies in South Asia have also highlighted the role of intergenerational power dynamics in affecting the autonomy of both women and men (Sen & Rastogi, 2006), including differences in female decision-making power between nuclear and extended households (Debnath, 2015, Mookerjee, 2017). A survey of pregnant, married women from our context showed in-laws made major financial decisions in 62% of joint households without the involvement of the junior couple (Gram et al., 2018).

On the whole, we expect our results to generalize most plausibly to rural households in the Plains of Nepal, North India, Pakistan and Bangladesh (Mandelbaum, 1993) – including the 33 million people in Nepal and North India who identify as Maithili – where the main structural parameters examined in our study have been replicated: patrilocal-patrilineal families, customary partition of the family estate when the junior couple moves out, seclusion norms stigmatizing women’s entry into public spaces, and large gender disparities in access to economic opportunities outside the household (Acharya and Bennett, 1983, Agarwal, 1994, Dyson and Moore, 1983, Mandelbaum, 1993).^11^ While the exact form of money management system observed in our study may not transfer verbatim to all such contexts, we still expect the patriarchal bargain to play a role in shaping intra-household negotiations over cash. The aforementioned structural features have also been observed in Newar and high-caste Hindu populations in the Hills of Nepal (Acharya and Bennett, 1983, Bennett, 1983), but caution should be applied transferring our results to Tibeto-Burman populations who differ significantly on female seclusion norms and inheritance rights (Acharya and Bennett, 1983, Agarwal, 1994).^12^,^13^

Anthropologists have long hypothesized that a shift from feudal land practices to competitive cash-based economies in South Asia would be accompanied by the break-up of the extended household, as sub-factions began to dispute the allocation of money or refuse to put their external earnings into a common household budget (Bailey, 1958, Epstein, 1962, Goode, 1963). Kandiyoti (1988) herself predicted a breakdown of the prevailing patriarchal bargain due to ‘the impact of new market forces, capital penetration in rural areas … or processes of chronic immiseration’ (p. 281). A survey of separated households in a rural village in Bangladesh found that 36% of respondents cited economic pressures as the most important cause of separation (Khuda, 1985). However, an analysis of nationally representative survey data from India from 1992 to 2006 revealed that the proportion of married women aged 15–29 residing in patrilocal extended households only decreased marginally from 64% in 1992–1993 to 56% in 2005–2006 (Allendorf, 2013).

Rather than view household separation over economic issues as a sign of the decline of the joint household institution, it seems more likely that such processes serve as an ‘escape valve’ for households where junior wives and husbands experience unbearable financial insecurity and exploitation in service of the patriarchal bargain. Nonetheless, while separated daughters-in-law often narrated their journey from daughter-in-law in a joint household to wife in a nuclear household as a story of emancipation and empowerment, the same daughters-in-law all held an expectation that they themselves would become mothers-in-law with attendant privileges over their own daughters-in-law in due time. At the same time, these daughters-in-law were now under the guardianship of their husbands, who have also been found in prior studies to potentially abuse their economic privileges (Chowbey, 2017, Kabeer, 1997, Singh and Bhandari, 2012).

In our sample, separated daughters-in-law sometimes downplayed power and control by their husband while accentuating such behavior by their mother-in-law. For example, they might initially state that no-one made decisions for them, but subsequently acknowledge – when prompted – that their husband had to be obeyed. Since the patriarchal bargain explicitly pits daughter-in-law against mother-in-law, while rendering men’s benefits from patriarchy invisible (Kandiyoti, 1988), our respondents may have been habituated to ‘seeing’ abuse by their mother-in-law and ‘unseeing’ abuse by their husband. Respondents in joint households may have considered it too ambitious to aspire to equality with their husbands, when they had not even freed themselves from their in-laws.^14^

Our study thus raises broader theoretical questions on the role of intergenerational power relationships in perpetuating patriarchy (Kandiyoti, 1988). The institution of household separation does not fundamentally challenge patriarchal norms mandating the subservience of junior women to senior women, nor does it alter expectations that women are subordinated to men in the household hierarchy. Instead, it may even perpetuate patriarchy through encouraging women to aim ‘for a change in the distribution of power, leaving intact the power structure itself’ (p. 81) (Irigaray, 1985), as junior women gain their autonomy at the expense of senior women’s access to financial protection (Vera-Sanso, 2005). True economic empowerment of South Asian women may require local women and men, activists, researchers and policy-makers to collectively reconsider the impact of intergenerational power relations on women’s control over cash.

## Footnotes

1 In-depth analyses of the determinants of female labor force participation, women’s rights to land or their control over durable marital assets lie outside the scope of this article, as these topics have already been treated extensively elsewhere (Agarwal, 1994; Bennett, 1992; Deere & Doss, 2006; Minturn & Kapoor, 1993).

2 Strictly speaking, Kandiyoti’s (1988) concept of a ‘patriarchal bargain’ applies outside the patriarchal belt as well, but in this article, we focus exclusively on the patriarchal bargain as defined for the patriarchal belt, a system of patriarchy that Kandiyoti (1988) called ‘classic patriarchy’.

3 In this article, we have largely refrained from drawing upon economic bargaining theory (Doss, 2013) as a framework for analyzing household dynamics. This is due to our focus on the ever-shifting intra-household *processes* of bargaining, negotiation and subversion (Kabeer, 1999) involved in realizing women’s agency. While the economic bargaining literature has uncovered numerous causal relationships between inputs into women’s fallback position (such as their income, wealth or educational status) and economic outcomes (such as household budget allocations on ‘female goods’), it has paid less attention to the agentic process of translating women’s resources into outcomes (Kabeer, 1999).

4 The term ‘financial guardian’ was invented for the purposes of this article to describe a relatively fixed set of roles and responsibilities related to the management of money in the Maithili household. None of the respondents themselves used the term describe a member of their household, although the word ‘guardian’ is in common use throughout the Plains of Nepal.

5 A substantial body of sociological literature on household money management in both the Global South (Singh & Bhandari, 2012; Kabeer, 1997) and Global North (Bennett, 2013) has sought to clarify the distinction between ‘control’ over money, which is ‘linked to the power to make major financial decisions or prevent discussion about these decisions’ (p. 47) (Singh & Bhandari, 2012) and ‘management’ of money, which refers to the practice of ‘organizing money in the household on a day-to-day basis’ (p. 47) (Singh & Bhandari, 2012). This distinction roughly corresponds to the difference in roles and responsibilities for a ‘household head’ and a ‘financial guardian’ in our context. However, after reviewing over 20 years’ of research into household money management, Bennett (2013) concluded that ‘a strict distinction between management and control may be hard to retain’ (p. 586). Indeed, a mother-in-law in our sample acting as a financial guardian might be able to take out a major loan without the explicit approval of the father-in-law due to her trusted role as a financial manager. At the same time, fathers-in-law might weigh in on the type of food that is bought and prepared and expect to be obeyed. Since our study was primarily concerned with intergenerational tensions over household money, the precise boundary between the duties of a mother-in-law vis-à-vis a father-in-law or a ‘household head’ vis-à-vis a ‘financial guardian’ is of lesser import: As compared to the senior in-laws, junior husbands and wives had usually *neither* the right to ‘manage’ *nor* the right to ‘control’ money in the household.

6 One concern is whether the mobility of daughters-in-law in our sample was unusually low due to their perinatal status. While many daughters-in-law and their guardians reported the presence of small children presenting an obstacle to their mobility, this was rarely the only factor. Family members overwhelmingly stated they denied daughters-in-law permission to go out in public due to a concern with breaching seclusion norms and daughters-in-law explicitly attributed their lack of mobility to such norms. Daughters-in-law were conversely able to move freely when this was socially acceptable. Thus, daughters-in-law frequently went to the market place in their natal village [daughter-in-law 6, 8, 10, Sahku; daughter-in-law 6, 7, Kote], if they had a suitable escort [daughter-in-law 1, 2, 4, Jamal; daughter-in-law 5, 8, Kote; daughter-in-law 5, 10, Sahku], or if the market was distant enough to remove the risk of running into neighbors [daughter-in-law 7, Kote; daughter-in-law 3, 7, Sahku]. While the perinatal status of our respondents may have played a role in making their mobility more challenging, these norm-based constraints on mobility likely persisted even after pregnancy and childbirth. In fact, a few respondents stated that carrying a small child facilitated mobility by legitimizing the presence of the daughter-in-law in public [daughter-in-law 7, Sahku; elder sister-in-law 3, Kote]. We thank an anonymous reviewer for highlighting this concern.

7 In three instances, daughters-in-law and mothers-in-law both reported having genuinely caring and supportive relationships with one another. In two of three instances, the family had recently undergone powerful possibly traumatic experiences, which could have triggered a sense of shared adversity and interdependence among family members. In one case, the husband had recently unexpectedly passed away at an early age [daughter-in-law 8, Kote]. In another instance [daughter-in-law 8, Sahku], the husband had formerly been kidnapped by Maoist insurgents who demanded a ransom for returning him. In two out of three instances, the incentives between daughter-in-law and mother-in-law were better aligned, since the husband’s income was not available to either of the two. As mentioned, one husband had passed away [daughter-in-law 8, Kote]. In a second case, the husband had ‘gone renegade’ and refused to share his income with any other family members [daughter-in-law 6, Sahku]. In all three cases, daughters-in-law felt no need to accumulate secret savings, because the care and support received from their mothers-in-law fulfilled their needs.

8 Even a 15-year-old son may be married to a girl due to the high prevalence of child marriage in our context (Raj, McDougal, & Rusch, 2012). It is also important to bear in mind that the number of years of marriage being reported by our respondents are likely inexact due to high rates of illiteracy in our population.

9 *Koseliya* is not to be confused with the Nepali word, *koseli,* which means ‘gift’. Eroglu also found that women’s secret kitties were so common in parts of Turkey that the colloquial term *vallah billah kesesi* (or ‘oath pouch’) was used for these (Eroglu, 2009). In parts of rural Bangladesh, women’s secret savings stores have also been referred to as *zolaitta* (Naved, 2000).

10 Note, the widespread nature of secret savings is perfectly consistent with the existence of strong disapproval of such practices by other household members. Empirically prevalent behavior does not have to be morally sanctioned according to local social norms (Bicchieri, Lindemans, & Jiang, 2014). For example, it is frequently argued that the existence of rampant corruption depends largely on whether people think others are corrupt as well, not on whether it is generally unacceptable or illegal (Köbis, van Prooijen, Righetti, & Van Lange, 2015).

11 In line with a considerable body of qualitative, ethnographic research (Baker et al., 2012, Small, 2009, Mookherji and LaFond, 2013) we use theory-based generalisation. We thus ask questions about whether the *core logic of our argument* transfers to other situations. Where the listed structural parameters are present, we can more plausibly expect our argument to transfer. Note, although foreign labor migration may have contributed to the observed power dynamics in our study, we excluded it from our list of structural parameters. Our core argument concerning the influence of the patriarchal bargain on junior women’s control over cash does not fundamentally hinge on the existence of male labor migration. In contexts with low levels of male labor migration, junior husbands may continue to have an incentive to ally with their wives over secret savings, if they are expected to give up control over earned income to senior household members.

12 Our findings contrast with Vera-Sanso’s (2005) study of low-caste, landless laborers in urban Tamil Nadu who reported junior couples from joint households declining their in-laws’ requests for financial support and declaring that husbands had a stronger duty towards care of their wives than their parents. Differences in context may account for this, as gender norms in North India have traditionally been considered more liberal than those in South India (Dyson & Moore, 1983; Mandelbaum, 1993; Bennett, 1983). Differences in economic context may also have played a role, as low-caste, landless laborers may have little inheritance property to offer as leverage over their own children.

13 Gender disparities in access to earning opportunities may have been exacerbated in our sample by the perinatal condition of daughters-in-law (see endnote 6); thus, women with older children may have had greater mobility. We are currently conducting a pilot study to design and collect quantitative indicators on women’s access to cash in the household that is embedded in a cohort study of mothers of newborn children over a period of six years. This study provides an opportunity for validating the generalizability of our results to a wider geographical context and for studying women with older children using complementary quantitative methods. In general, future quantitative researchers may be able to create new revealed-preference models for intra-household bargaining (Doss, 2013) or new lab-in-the-field experiments to elicit preferences for hiding money (Said, Mahmud, d'Adda, & Chaudhry, 2017) inspired by our results.

14 A question arises as to whether an alliance with the husband constitutes empowerment or dependency. The dividing line between ‘social support’ and ‘dependency’ has long been a contentious issue in feminist literature. Independence may protect from perceived risks of exploitation and conflict (Kabeer, 2001), as social dependence requires agents to continually ingratiate themselves with their supporter to extract favors (Iversen, 2003). On the other hand, ‘autonomy fetishism’ (Khader, 2015) may discourage experiences of affiliation, communion, and cooperation (Riger, 1993) and impose unrealistic burdens of self-sufficiency (Sen, 1985). We navigated this tension by paying close attention to women’s own explanations and descriptions of their actions so as to distinguish ‘legitimate’ social support from ‘illegitimate’ interference and control (Gram et al., 2016). These accounts invariably portrayed alliances with husbands in a positive light, but daughters-in-law may have been biased towards favoring dependency on their husband over dependency on their in-laws. The nature of self-reported, qualitative data makes it almost impossible to distinguish women’s ‘subjective’ experience of oppression from their ‘objective’ (Jónasdóttir, 1988) level of empowerment. Observational ethnographic methods may help explore differences between reported and actual behavior, but the perceptual, emotional and cognitive components of human relationships will remain intrinsically subjective. We thank an anonymous reviewer for this question).
